# The protective effects of hepatocyte growth factor on the intestinal mucosal atrophy induced by total parenteral nutrition in a rat model

**DOI:** 10.1007/s00383-021-05002-0

**Published:** 2021-08-26

**Authors:** Koshiro Sugita, Tatsuru Kaji, Keisuke Yano, Makoto Matsukubo, Ayaka Nagano, Mayu Matsui, Masakazu Murakami, Toshio Harumatsu, Shun Onishi, Koji Yamada, Waka Yamada, Mitsuru Muto, Kotaro Kumagai, Akio Ido, Satoshi Ieiri

**Affiliations:** 1 Department of Pediatric Surgery, Medical and Dental Area, Research and Education Assembly, Research Field in Medical and Health Sciences, Kagoshima University, 8-35-1, Sakuragaoka, Kagoshima, 890-8520 Japan; 2 Clinical Training Center, Kagoshima University Hospital, Kagoshima, Japan; 3 Digestive and Lifestyle Diseases, Department of Human and Environmental Sciences, School of Medical and Dental Sciences, Kagoshima University Graduate, Kagoshima, Japan

**Keywords:** Total parenteral nutrition, Intestinal mucosal atrophy, Hepatocyte growth factor (HGF)

## Abstract

**Purpose:**

Total parental nutrition (TPN) causes gastrointestinal mucosal atrophy. The present study investigated the effects of hepatocyte growth factor (HGF) on the intestinal mucosal atrophy induced by TPN.

**Methods:**

Rats underwent jugular vein catheterization and were divided into four groups: oral feeding (OF), TPN alone (TPN), TPN plus low-dose HGF (0.3 mg/kg/day; TPNLH), and TPN plus high-dose HGF (1.0 mg/kg/day; TPNHH). On day 7, rats were euthanized, and the small intestine was harvested and evaluated histologically. The expression of c-MET, a receptor of HGF, and nutrition transporter protein were evaluated using quantitative polymerase chain reaction.

**Results:**

The jejunal villus height (VH) and absorptive mucosal surface area in the TPNHH group were significantly higher than in the TPN group (*p* < 0.05). The VH in the ileum showed the same trend only in the TPNHH group, albeit without statistical significance. The crypt cell proliferation rate (CCPR) of the jejunum in both HGF-treated groups was significantly higher than in the TPN group (*p* < 0.01). The expression of c-MET and transporter protein in all TPN-treated groups was decreased compared with that in the OF group.

**Conclusion:**

HGF attenuated TPN-associated intestinal mucosal atrophy by increasing the villus height, which was associated with an increase in CCPR.

## Introduction

Parenteral nutrition is a crucial therapeutic modality for various diseases in neonates, children, and adults [1]. Nutrition therapy for children is essential for not only life support but also growth and development. Thus, patients who are unable to receive oral feeding due to gastrointestinal dysfunction or morphological disorders often require total parenteral nutrition (TPN). However, TPN causes various complications, such as gastrointestinal mucosal atrophy and intestinal failure-associated liver disease (IFALD) [2, 3], including hepatic steatosis, cholestasis, and liver fibrosis [4–6].

Our previous study using a TPN rat model or a short bowel syndrome rat model with or without bowel resection showed that both ghrelin [7] and glucagon-like peptide-2 (GLP-2) [8] attenuated intestinal mucosal atrophy and IFALD [9, 10]. However, the effect of ghrelin on intestinal mucosal atrophy was less marked than that of GLP-2, and a high dose of GLP-2 deteriorated the parenteral nutrition-associated liver disease (PNALD). Since both agents have their advantages and disadvantages clinically, we have been seeking more effective agents than ghrelin and GLP-2.

Hepatocyte growth factor (HGF) was first purified and isolated as a potent hepatocyte mitogen from the plasma of patients with fulminant hepatic failure [11]. HGF and its receptor c-MET exert mitogenic and monogenic activities in not only hepatic tissue but also digestive tissues [12]. Many studies have reported that HGF includes physiologically active peptides with multiple functions, such as anti-inflammation, tissue repair, and anti-apoptosis [13–16].

The present study investigated the effects of HGF on TPN-associated intestinal mucosal atrophy using a rat model. First, we revealed the histological changes on the intestine by administration of HGF. Second, we evaluated some endpoints regarding the mechanism on the proliferation of intestinal mucosa by administration of HGF. Third, we suggested signal process on the effects of HGF.

## Materials and methods

### Animals

Eight-week-old male Sprague–Dawley (SD) rats of 250–280 g in body weight (Kyudo Co., Ltd., Saga, Japan) were used in this research. The rats were individually housed in metabolic cages with ad libitum access to standard rat chow and water and were acclimatized to their environment for seven days before the experiments. The rats were maintained under standardized temperature (23 °C ± 1 °C) and humidity (50% ± 10%) with a 12-h light–dark cycle (lights on at 7:00 a.m.).

All of the experimental procedures were approved by the Laboratory Animal Committees of Kagoshima University Graduate School and were performed in accordance with the “Guidelines for the Care and Use of Laboratory Animals” (Approval number: MD20014).

### Study design

A previous study regarding hepatic tissue reported that the intravenous administration of recombinant human-HGF (rh-HGF) (0.3 mg/kg/day) to rats once a day was effective for treating cirrhosis, while rh-HGF (1.0 mg/kg/day) caused adverse effects [17]. We therefore defined the intravenous administration of 0.3 mg/kg/day as a “low dose” and 1.0 mg/kg/day as a “high dose”. Rh-HGF (Eisai Co., Ltd., Tokyo, Japan) [18] was dissolved in phosphate-buffered saline and administered by intravenous injection once a day via the placement of a central venous catheter. All rats were divided into 4 groups as follows (*n* = 10 per groups): oral feeding (OF group), TPN alone (TPN group), TPN plus low-dose HGF (0.3 mg/kg/day; TPNLH group), and TPN plus high-dose HGF (1.0 mg/kg/day; TPNHH group). On day 7, the rats were anesthetized, weighed, and killed. The gross intestinal morphology was assessed, and tissue was harvested for subsequent analyses.

### The surgical procedure and maintenance methods

All rats were anesthetized with isoflurane (1.5% inhalation by mask) and underwent catheterization by the cut-down method through the jugular vein. A silastic catheter with an outside diameter of 0.3 × 0.8 mm (NISHINKIKAICO., Ltd., Tokushima, Japan) was used, tunneled out of the back, and attached to a standard swivel device (LOMIR BIOMEDICAL INC., Quebec, Canada). The procedures were performed with the aid of an operating microscope. All rats received cefazolin (50 mg/kg per dose, subcutaneously; Otsuka Pharmaceutical Factory, Inc., Tokushima, Japan) to prevent postoperative infection and buprenorphine (0.01 mg/kg per dose, subcutaneously; Otsuka Pharmaceutical Co., Ltd.) for analgesia. They were allowed ad libitum access to water immediately after surgery.

TPN was delivered by a multichannel syringe pump (KDS Legato 200 Series Syringe Pump Series; KD Scientific, Inc., Holliston, MA, USA). After catheterization, the rats were maintained with low-concentration NEOPAREN^®^ No. 2 (Otsuka Pharmaceutical Co., Ltd.) TPN solution (60 mL/day), to which 20% Intralipos^®^ (Otsuka Pharmaceutical Co., Ltd.) had been added. The composition of the TPN solution was as follows (in g/L): amino acids 25, dextrose 145, and soybean oil 33.3. The solution also contained the following electrolytes (final mmol/L): 41.6 Na^+^, 22.5 K^+^, 41.6 Cl^−^, 4.1 Ca^2+^ and 4.1 Mg^2+^. After 24 h, the composition of the TPN solution was switched to the following (in g/L): amino acids 31.6, glucose 203, and soybean oil 33.3, with similar electrolyte additives. The TPN solution was delivered at a rate of 60 mL/day. This provided equivalent isocaloric/isonitrogenous nutritional support to all TPN-fed rats, consisting of 76.4 kcal/rat/day (1.9 g protein, 2.0 g fat, and 12.2 g carbohydrate). The rats in the OF group had the amount of chow they were served adjusted to provide the same calories as TPN-fed rats.

On day 7, all rats were anesthetized by isoflurane inhalation. Blood was obtained from the heart and immediately centrifuged at 1500×*g* for 15 min at 4 °C. All serum samples were stored at − 80 °C until use. After blood collection, the rats were euthanized by exsanguination.

### The intestinal morphology and histology

The total small intestine, from the ligament of Treitz to ileocecal valve, was harvested for the gross and microscopic morphological analyses. The mesentery was removed, and samples for the microscopic analysis were harvested from the jejunum (5.0 cm below the ligament of Treitz) and the distal ileum (5.0 cm above the ileocecal valve). Each sample was quickly opened along the mesenteric border, rinsed in cold saline, and weighed. Subsequently, each sample fixed in a 10% formaldehyde neutral buffer solution for 24 h. Paraffin sections of formalin-fixed tissue were cut at a thickness of 3 μm and stained with hematoxylin and eosin. For each sample slide, microscopic measurements of the villus height, villus width, crypt depth, and the muscle layer from 10 well-oriented villi/crypt units were made. Quantification was performed with the help of an expert pathologist. The absorptive mucosal surface area per 1 cm^2^ of intestine was calculated using previously described methods. In brief, the mucosal surface area was calculated by first considering the intestine as a cylinder and then multiplying the additional mucosal surface area contributed by the villi, with each villus considered a cone [19].

### Crypt cell proliferation

The crypt cell proliferation rate (CCPR) was quantified by immunohistochemistry using Ki-67 (Cell Signaling Technology, Inc., Denver, MA, USA) as a marker of active cell division, as previously described [20]. In brief, antigen retrieval was performed by boiling the tissue sections in 0.01 M citrate buffer at pH 6. After the blocking of endogenous peroxidase activity and nonspecific antigen binding, the tissue sections were incubated with anti-histone Ki-67 overnight in a moist chamber. The appropriate dilution of Ki-67 was 1:400. After being washed in Tris buffer saline, the tissue sections were incubated with universal secondary antibody (Signal Stain® Boost IHC Detection Reagent; Cell Signaling Technology, Inc.). Immune detection was performed using diaminobenzidine as chromogen and hydrogen peroxide, followed by counterstaining with hematoxylin. The CCPR was calculated as the number of Ki-67-positive cells present among 10 consecutive well-oriented crypts per slide.

### Real-time quantitative polymerase chain reaction (qPCR) of c-MET, SGLT-1, GLUT2, and GLUT5 in the jejunum tissue

To evaluate nutrient absorption, we measured the expression of SGLT-1, GLUT2, and GLUT5 using real-time qPCR in just the jejunum tissue. The tissue of the jejunum was evaluated because the main mucosal morphological alterations were recognized in the jejunum. In addition, we evaluated the expression of c-MET to investigate the mechanism of the HGF. The first-standard cDNA was synthesized using SuperScript IV^®^ Reverse Transcriptase (Thermo Fisher Scientifics, Waltham, MA, USA) with oligo (dT) primer. Each cDNA sample was then diluted with RNase/DNase-free water to 1.25 ng template RNA/μL. The expression of each gene was analyzed by qPCR using the Bio-Rad CFX96 system (BioRad Laboratories, Inc., Hercules, CA, USA). Standard DNA was generated by block double-stranded DNA fragments synthesis (Integrated DNA Technologies, Inc., Skokie, IL, USA).

A housekeeping gene was used peptidyl-prolyl *cis*–*trans* isomerase A (PPIA) as oligo (dT) primer, RPr PPIA F1: 5′-ATACAGGTCCTGGCATCTTGTCCAT-3′ (forward), RPr PPIA R1: 5′-CTTCTTTCACCTTCCCAAAGACCAC-3′ (reverse). The other primers used for each sample were as follows: RPr c-MET F1; 5′-ACCTCAGCAA TGTCAGCACCA-3′ (forward), RPr c-MET R1; 5′-GGCCATGTGATGTCATTCTGG-3′ (reverse) [21], RPr SGLT-1 F1; 5′-CCAAGCCCATCCCAGACGTACACC-3′ (forward), RPr SGLT-1 R1; 5′-CTTCCTTAGTCATCTTCGGTCCTT-3′ (reverse) [22], RPr GLUT2 F1; 5′-TTTGCAGTAGGCGGAATGG-3′ (forward), RPr GLUT-2 R1; 5′-GCCAACATGGCTTTGATCCTT-3′ (reverse) [22], and RPr GLUT-5 F1; 5′-TGCAGAGCAACGATGGAGAAA-3′ (forward), RPr GLUT-5 R1; 5′-ACAGCAGCGTCAGGGTGAAG-3′ (reverse) [22]. These measurements were performed by Repertoire Genesis Inc., Osaka, Japan.

### Statistical analyses

The data were presented as the mean values ± standard error (SE). Gene expression using real-time quantitative polymerase chain reaction was presented as the ratio to the housekeeping gene. Statistical analyses between groups and time courses were performed using a one-factor analysis of variance (ANOVA) followed by Tukey's multiple-comparison post hoc test. *p* values of < 0.05 were considered to indicate statistical significance.

All statistical analyses were performed with EZR (Saitama Medical Center, Jichi Medical University, Saitama, Japan), which is a graphical user interface for R (The R Foundation for Statistical Computing, Vienna, Austria). More precisely, it is a modified version of R commander designed to add statistical functions frequently used in biostatistics [23].

## Results

### The microscopic intestinal morphology in the jejunum

**Fig. 1:**
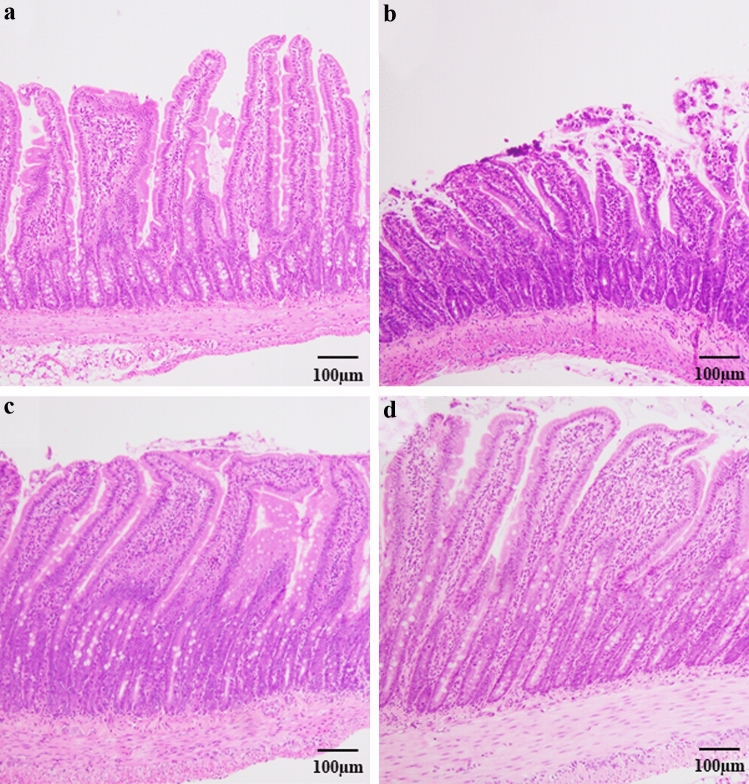
Hematoxylin–eosin stain (× 100) for jejunum. **a** Oral feeding (OF), **b** TPN alone (TPN), **c** TPN plus low-dose HGF (TPNLH), **d** TPN plus high-dose HGF (TPNHH)

A representative image of the histological morphology in the jejunum is shown in Fig. 1. The TPN group (Fig. 1b) showed more severe mucosal atrophy than the OF group (Fig. 1a). The TPNLH (Fig. 1c) and TPNHH groups (Fig. 1d) showed attenuated mucosal atrophy in the jejunum.

**Fig. 2:**
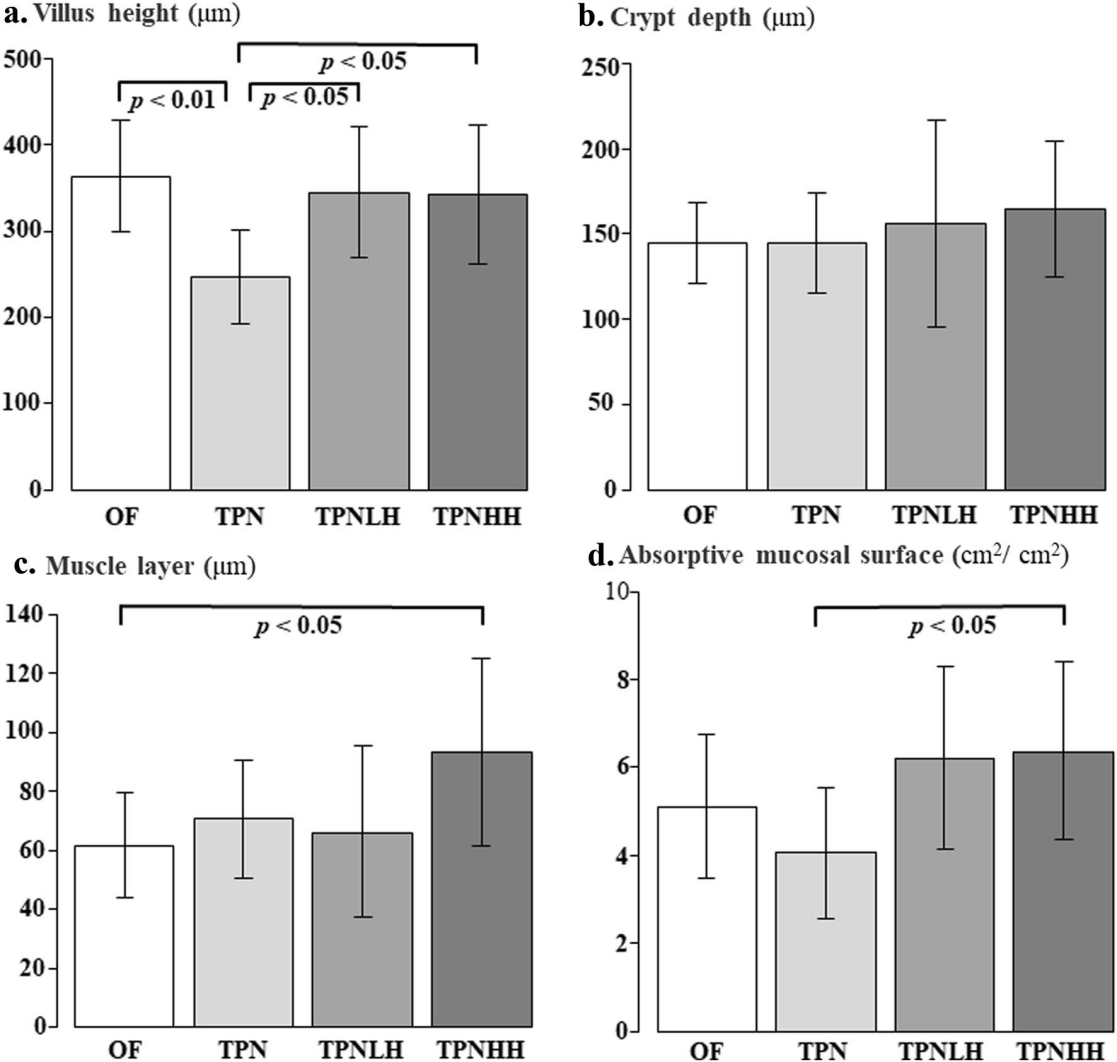
Multiple comparisons of the jejunal morphology. *OF* oral feeding, *TPN* TPN alone, *TPNLH* TPN plus low-dose HGF, *TPNHH* TPN plus high-dose HGF. **a** Villus height (μm), **b** crypt depth (μm), **c** muscle layer (μm), **d** absorptive mucosal surface (cm^2^/cm^2^). The presence or absence of a significant difference is indicated by a black bar, and the *p* value is shown below it

The villus height in the TPN group was significantly lower than that in the TPNLH group (*p* = 0.018) and TPNHH group (*p* = 0.021) (Fig. 2a). 7-day TPN thus induced mucosal atrophy in the jejunum. There were no significant differences in the crypt depth among groups (Fig. 2b). The muscle layer in the TPNHH group was significantly thicker than that in the OF group (*p* = 0.041) (Fig. 2c). The absorptive mucosal surface in the TPNHH group was significantly higher than that in the TPN groups (*p* = 0.011) (Fig. 2d).

### The microscopic intestinal morphology in the ileum

**Fig. 3:**
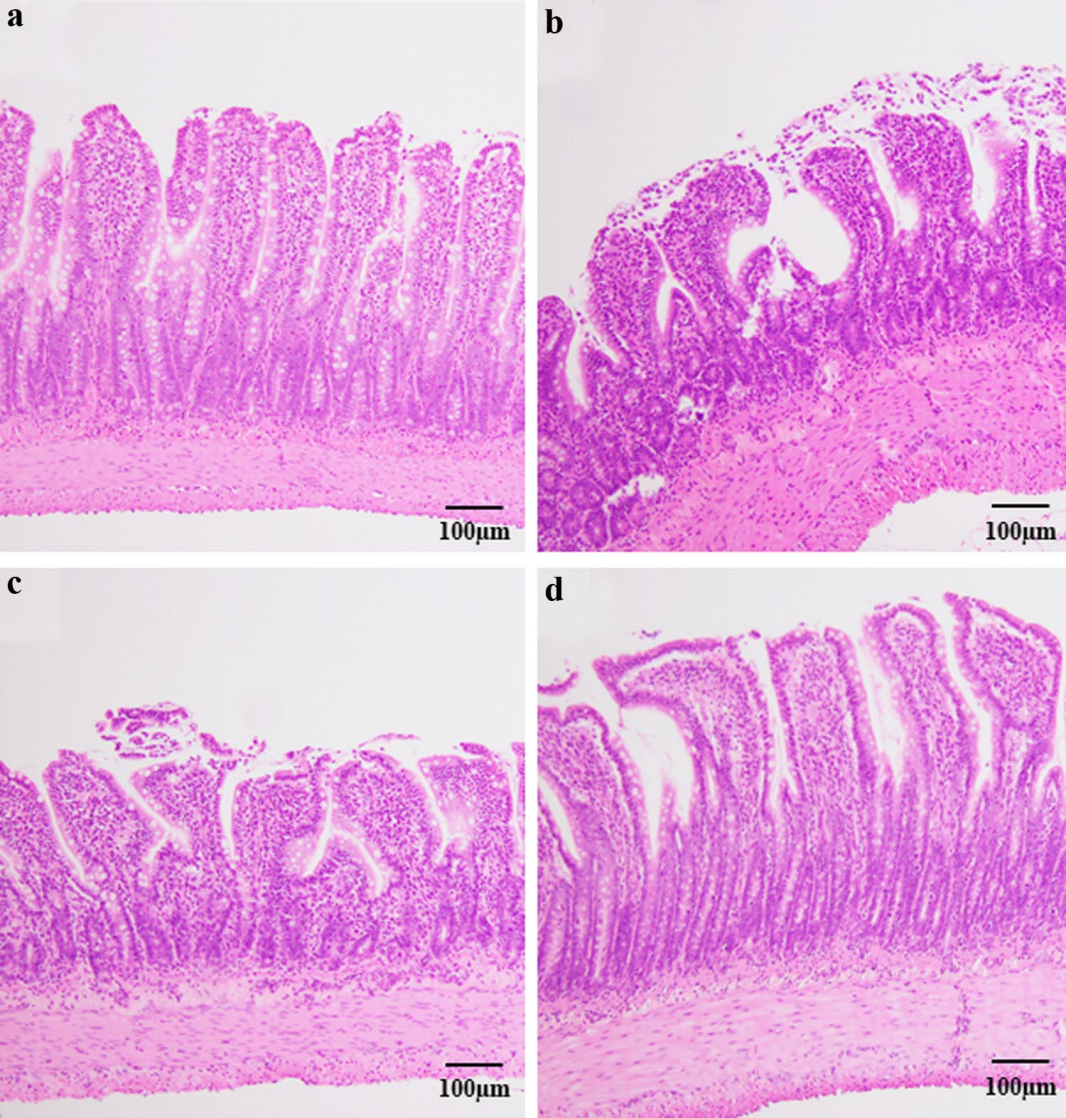
Hematoxylin–eosin stain (× 100) for ileum. **a** Oral feeding (OF), **b** TPN alone (TPN), **c** TPN plus low-dose HGF (TPNLH), **d** TPN plus high-dose HGF (TPNHH)

A representative image of the histological morphology in the ileum is shown in Fig. 3. The TPN group showed mild mucosal atrophy compared to the OF group (OF group: Fig. 3a, TPN group: Fig. 3b). The TPNLH group did not show attenuated mucosal atrophy in the ileum (Fig. 3c), whereas the TPNHH group did show attenuated mucosal atrophy (Fig. 3d).

**Fig. 4:**
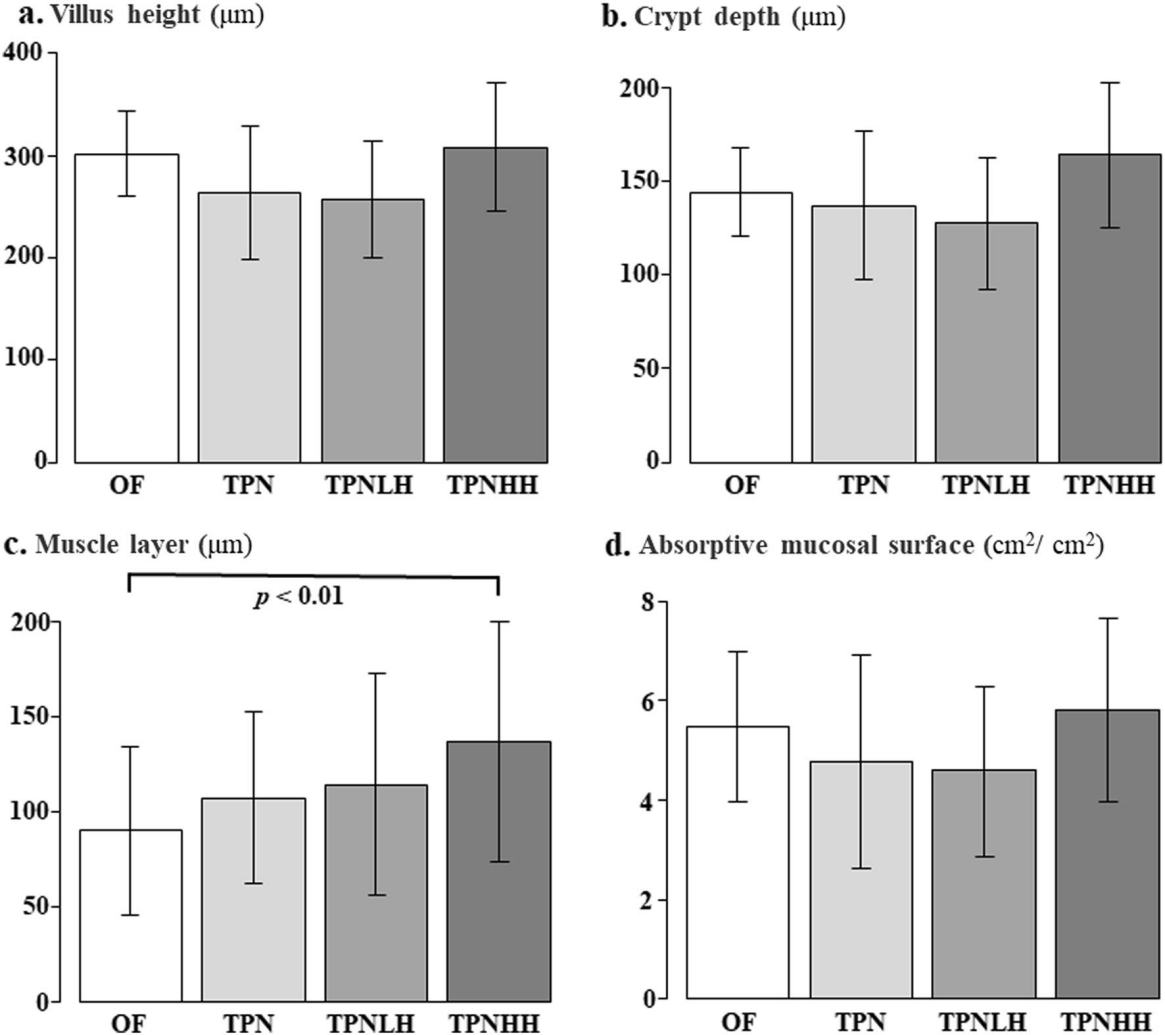
Multiple comparisons of the ileal morphology. *OF* oral feeding, *TPN* TPN alone, *TPNLH* TPN plus low-dose HGF, *TPNHH* TPN plus high-dose HGF. **a** Villus height (μm), **b** crypt depth (μm), **c** muscle layer (μm), **d** absorptive mucosal surface (cm^2^/cm^2^). The presence or absence of a significant difference is indicated by a black bar, and the *p* value is shown below it

The villus height and crypt depth showed no significant difference among the groups (Fig. 4a, b). The muscle layer in the TPNHH group was significantly thicker than that in the OF group (*p* < 0.01) (Fig. 4c). There was no significant difference in the absorptive mucosal surface among the groups (Fig. 4d).

### Crypt cell proliferation rates in the jejunum

**Fig. 5:**
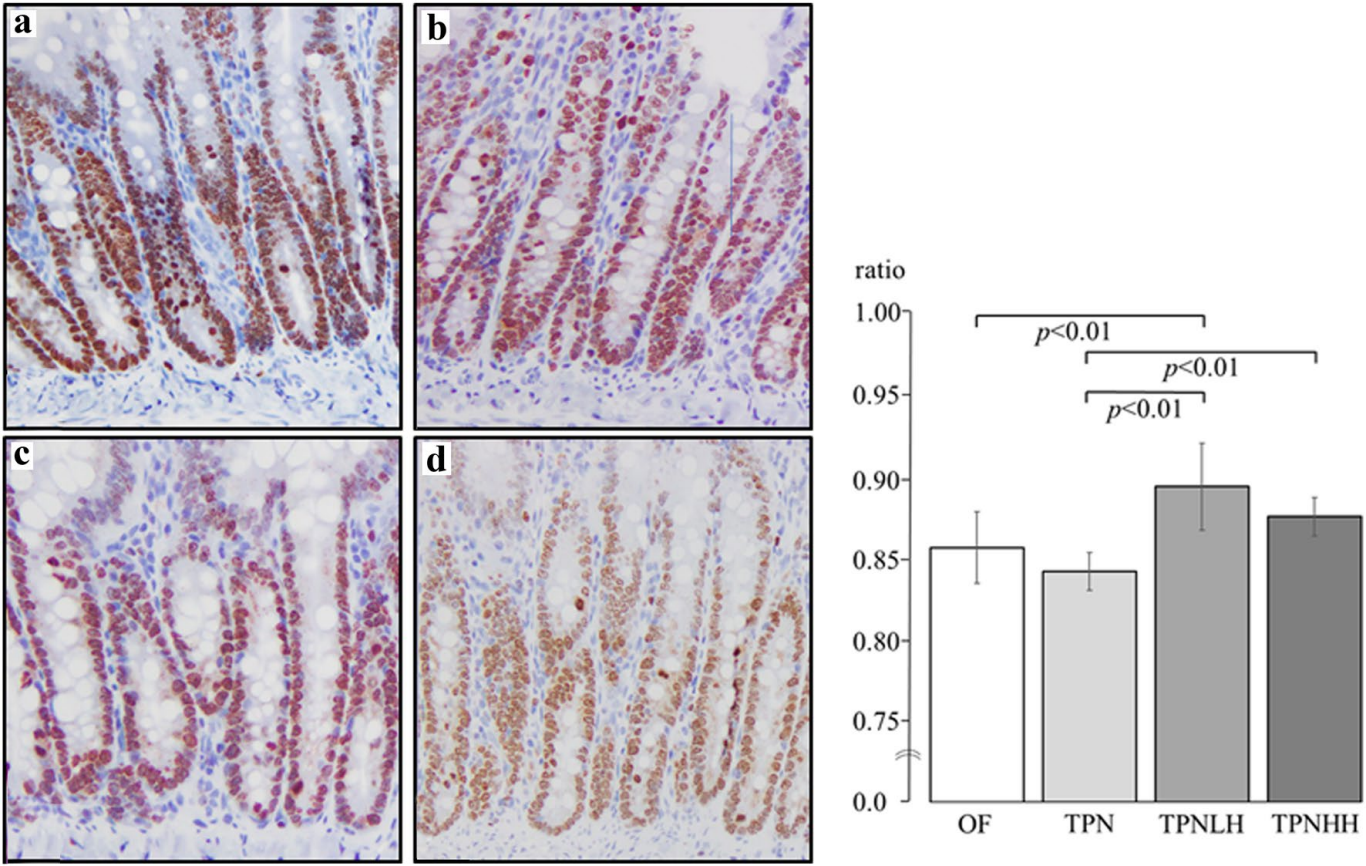
Immunohistochemical stain (× 200) by Ki-67 for jejunum. **a** Oral feeding (OF), **b** TPN alone (TPN), **c** TPN plus low-dose HGF (TPNLH), **d** TPN plus high-dose HGF (TPNHH). The presence or absence of a significant difference is indicated by a black bar, and the *p* value is shown below it

The CCPR in the TPNLH and TPNHH groups was significantly higher than that in the TPN group (TPN 0.84 ± 0.01 vs. TPNLH 0.90 ± 0.03 and TPNHH 0.88 ± 0.01, all *p* < 0.01). In addition, the CCPR in the TPNLH group was significantly higher than that in the OF group (*p* < 0.01) (Fig. 5).

### Crypt cell proliferation rates in the ileum

**Fig. 6:**
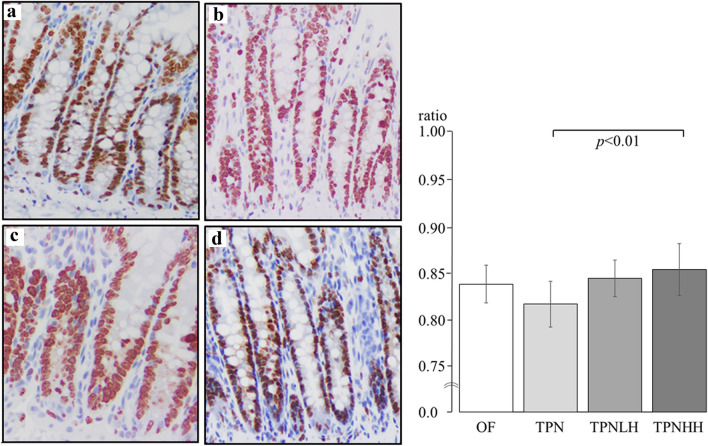
Immunohistochemical stain (× 200) by Ki-67 for ileum. **a** Oral feeding (OF), **b** TPN alone (TPN), **c** TPN plus low-dose HGF (TPNLH), **d** TPN plus high-dose HGF (TPNHH). The presence or absence of a significant difference is indicated by a black bar, and the *p* value is shown below it

The CCPRs were 0.84 ± 0.02 in the OF group, 0.82 ± 0.03 in the TPN group, 0.84 ± 0.02 in the TPNLH, and 0.85 ± 0.03 in the TPNHH group. Only the CCPR in the TPNHH group was significantly higher than that in the TPN group (*p* < 0.01) (Fig. 6).

### The expression of c-MET in the jejunum according to qPCR

**Fig. 7:**
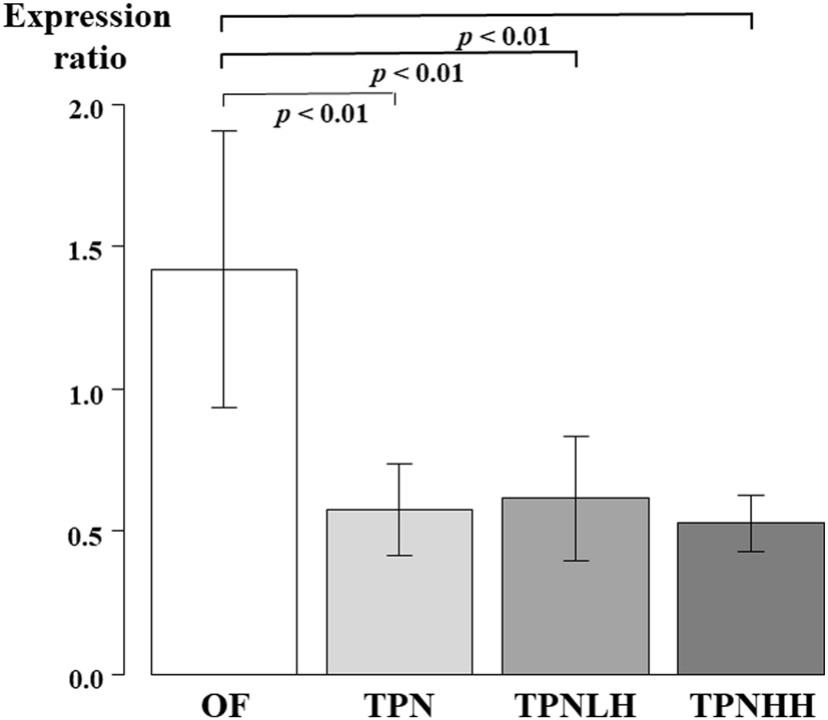
The expression of c-MET in the jejunum. *OF* oral feeding, *TPN* TPN alone, *TPNLH* TPN plus low-dose HGF, *TPNHH* TPN plus high-dose HGF. The presence or absence of a significant difference is indicated by a black bar, and the *p* value is shown below it

The expression of c-MET was significantly higher in the OF group than in the other groups (OF vs. TPN, TPNLH and TPNHH: all *p* < 0.01) (Fig. 7). There were no significant differences in the c-MET expression among the three TPN-treated groups.

### The expression of nutrition transporter proteins

**Fig. 8:**
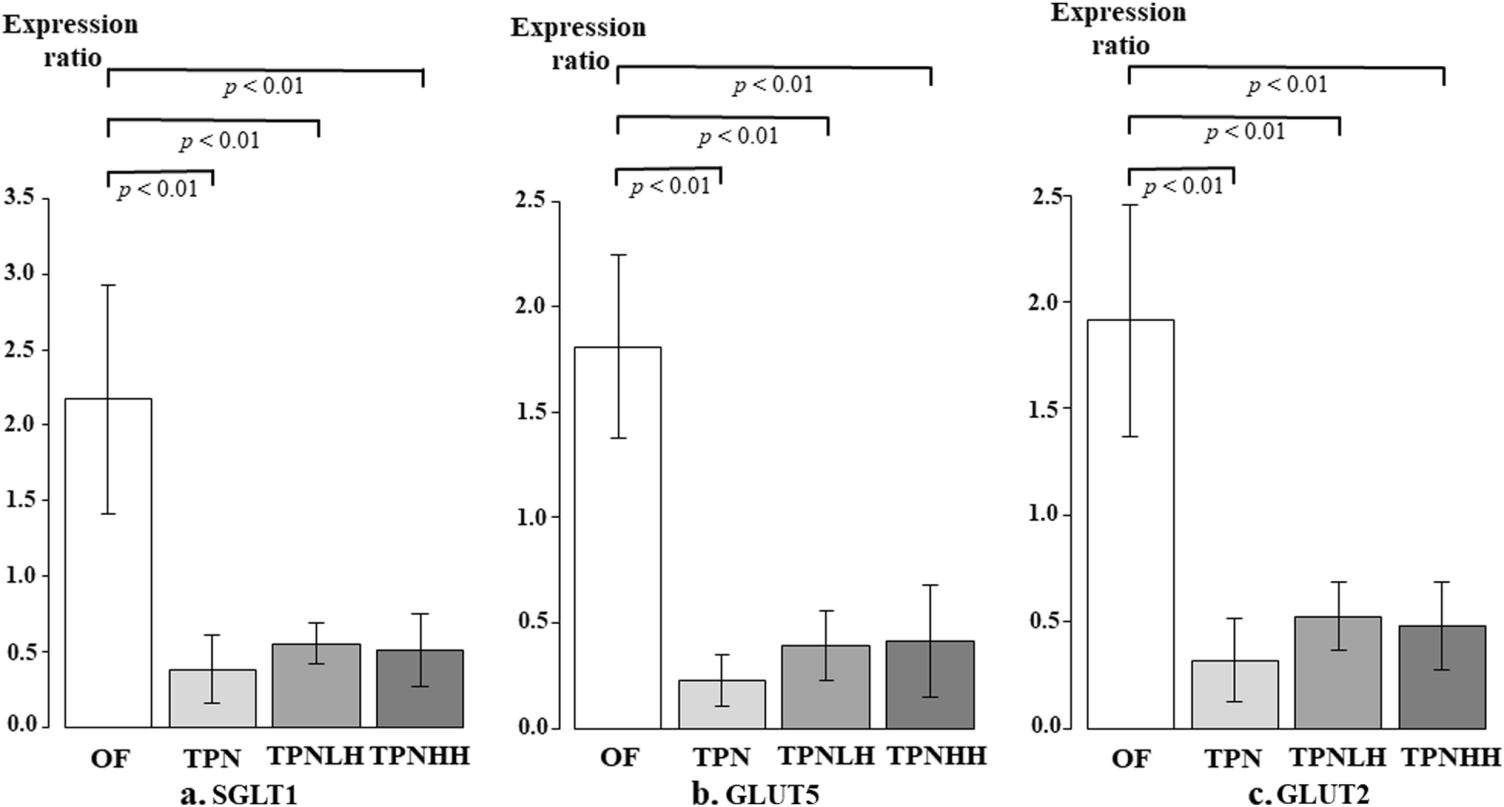
The expression of nutrition transporter in the jejunum. *OF* oral feeding, *TPN* TPN alone, *TPNLH* TPN plus low-dose HGF, *TPNHH* TPN plus high-dose HGF. **a** SGLT1, **b** GLUT5, **c** GLUT2. The presence or absence of a significant difference is indicated by a black bar, and the *p* value is shown below it

The expression of nutrition transporter proteins, such as SGLT-1 (Fig. 8a), GLUT5 (Fig. 8b), and GLUT2 (Fig. 8c) in the OF group was significantly higher than in the other groups (*p* < 0.01).

## Discussion

We have been exploring new agents to prevent intestinal mucosal atrophy induced by TPN. In our resent study on hepatic steatosis induced by TPN using rats, the administration of HGF was effective [24]. Furthermore, HGF modulates intestinal epithelial cell proliferation and migration, serving as a critical regulator of intestinal wound healing [25], so we focused on this agent in the present study.

The major findings in this study were as follows: (1) the jejunal villus height in the HGF-treated groups was significantly greater than that in the TPN group; (2) the absorptive mucosal surface area in the HGFHH group was significantly greater than that in the TPN group; (3) muscular layer in both the jejunum and ileum was in the TPNHH group significantly greater than that in the OF group; (4) the CCPR in the TPNLH and TPNHH group was significantly higher than that in the TPN group; and (5) the expression of c-MET and nutrition transporters in the OF group was significantly higher than in the other three groups.

Some previous studies have shown that just 5- to 7-day TPN can reduce villous height, crypt depth, and CCPR as TPN-associated mucosal atrophy [7, 26]. Regarding the morphological change in this study, we found that both jejunal and ileal mucosal atrophy were induced by 7-day TPN in a rat model. First, in the jejunal mucosal morphological evaluation, the villus height in both the HGF-treated groups and calculated absorptive mucosal area in the TPNHH group was significantly higher than that in the TPN group. We clarified that the CCPRs in both HGF-treated groups were significantly increased compared with the TPN group. We believe this was why we observed the preservation of the villus height. Second, in the ileal mucosal morphological evaluation, the villus height and crypt depth and absorptive mucosal area in the TPNHH group tended to be higher than in the TPN group, albeit without a significant difference. Since the CCPR in the TPNHH group was significantly higher than that in the TPN group, we suspected that HGF had an effect of elongating the ileal mucosa, as neither such low- nor high-dose HGF would be sufficient to increase the amount of ileal mucosa and absorptive mucosal area. Further studies will be required to determine the appropriate dose of HGF.

The expression of c-MET, which is the receptor of HGF, in the OF group was significantly higher than that in the other three groups. Tahara et al. showed that c-MET phosphorylation was increased in the colonic mucosa upon the administration of HGF using an experimental ulcerative colitis rat model [25]. Given the histological findings, we expected that the expression of the c-MET in the HGF-treated groups would be increased compared with that in the TPN group, but the obtained results showed the same levels between the HGF-treated groups and the TPN group. Timmapuri et al. examined the glucagon immunoreactivity in the small intestine induced by the administration of HGF using a 70% bowel resection rat model. They found that the glucagon immunoreactivity was increased only in the jejunum, not the ileum, by HGF administration. Since glucagon is spliced from proglucagon and produced in the L cells of the small intestine as a growth factor, they speculated that HGF affected the mucosal proliferation indirectly through the activation of glucagon in the small intestine [27]. This report was compatible with the different findings in jejunal and ileal results in our study. Therefore, we considered that the process on the effect of HGF might related to glucagon.

The expression of nutrition transporter proteins, such as SGLT1, GLUT2, and GLUT5, also showed a significant decrease in the TPN and both HGF-treated groups compared to the OF group. Kato et al. found that the administration of HGF induced the upregulated expression of SGLT1 and GLUT5 [28] in a rat model of massive short-bowel resection [29]. In the present study, the expression of the nutrition transporter proteins tended to be higher in the HGF-treated groups than in the TPN group, but the expression did not reach that in the OF group. These results showed that TPN caused not only morphological changes but also decreased absorption capacity and reminded us that oral feeding and enteral nutrition are important for both the gastrointestinal morphology and function.

To our knowledge, muscle layer in the small intestine following the administration of HGF has not been confirmed from previous studies. Since HGF has proliferative and mitotic effects, it would not be bizarre for the intestinal mucosa to be thickened by the administration of HGF. The augmentation of the muscle layer by the administration of HGF may be induced by indirect effect through the activation of glucagon, as described above in Timmapuri’s study [27].

In this study, the HGF dose was set at 0.3 mg/kg/day for the low dose and 1.0 mg/kg/day for the high dose. However, while a sufficient effect was obtained in the low-dose group in the jejunum, the same effect was obtained only in the ileum in the high-dose group. In a previous study, the systemic administration of 1.0 mg/kg/day was reported to have adverse effects on the liver. In addition, luminal administration of HGF via the jejunum has been shown to be as effective as systemic administration [30, 31]. Further studies are needed to clarify the mechanism and determine the appropriate dose of HGF and the manner in which it should be administered in order for it to be used in the clinical setting.

Several limitations associated with the present study warrant mention. First, the amount of HGF to be administered was set by referencing the liver dose, not the gastrointestinal tract. Second, the expression in the ileum was not analyzed due to a lack of morphologically significant results.

## Conclusions

The administration of HGF improved TPN-associated intestinal mucosal atrophy by increasing the villus height. We clarified that one of the mechanisms underlying these results involved an increase in the CCPR of the intestine. However, further studies are needed to clarify the mechanism underlying the effects of HGF on TPN-induced intestinal mucosal atrophy.
